# Applying a punch with microridges in multistage deep drawing processes

**DOI:** 10.1186/s40064-016-3371-2

**Published:** 2016-10-06

**Authors:** Bor-Tsuen Lin, Cheng-Yu Yang

**Affiliations:** Department of Mechanical and Automation Engineering, National Kaohsiung First University of Science and Technology, 2 Juoyue Road, Nantz District, Kaohsiung, Taiwan, ROC

**Keywords:** Aspect ratio, Finite element method, Microridge, Multistage deep drawing

## Abstract

The developers of high aspect ratio components aim to minimize the processing stages in deep drawing processes. This study elucidates the application of microridge punches in multistage deep drawing processes. A microridge punch improves drawing performance, thereby reducing the number of stages required in deep forming processes. As an example, the original eight-stage deep forming process for a copper cylindrical cup with a high aspect ratio was analyzed by finite element simulation. Microridge punch designs were introduced in Stages 4 and 7 to replace the original punches. In addition, Stages 3 and 6 were eliminated. Finally, these changes were verified through experiments. The results showed that the microridge punches reduced the number of deep drawing stages yielding similar thickness difference percentages. Further, the numerical and experimental results demonstrated good consistency in the thickness distribution.

## Background

The forming processes of sheet metals are advantageous for increasing the rate of production, decreasing the processing costs, improving the quality, and enhancing the yields of mechanical products and are thus widely applied in automotive manufacturing and various industrial products (i.e., electronic, information, and communication products). Recently, industries have emphasized product miniaturization, in which strength and reliability must be considered. This converts the forming processes of thin sheet metals into potential technologies for micro/meso component production technologies.

During microforming, the grain size of the drawing material affects the forming properties in reduced component dimensions; this is referred to as the size effect (Armstrong 1961). Several published articles have described endeavors to improve the drawability of the micro deep drawing process. Erhardt et al. (1999) proposed the concept of warm microforming processes using an Nd/YAG laser to heat sheet metal workpieces locally, thereby reducing the drawing force and increasing the formability. Yagami et al. (2007) utilized a cylindrical cup drawing experiment to investigate the influence of controlling the blank holder motion on removing wrinkles and improving drawability.

In the drawing process, the sheet metal and punch are in close contact with each other when entering the die cavity. During this process, the sheet metal slides through the blank holder and lower die surface. Therefore, friction must be increased between the sheet metal and punch or decreased between the metal sheet and blank-holder or lower die to improve formability (Gong et al. 2011). Gong et al. (2011) used a diamond-like carbon coated blank holder and lower die in a cylindrical cup microforming experiment to reduce friction between the metal sheet and lower die and the blank holder, thereby enhancing formability. Additionally, the micro-features can be used to change the physical properties of the contact surface of the material (Segu and Kim 2014). Steinhoff et al. (1996) investigated the use of deterministic and stochastic surface structures in strip drawing processes. The experimental result shows that the hydrostatic lubrication pockets results in a considerable improvement of the tribological behaviour. Costa and Hutchings (2009) studied the friction effects of controlled surface patterning in tribological situations by using the strip drawing test. The result shows that the relative orientation between the pattern and the drawing direction strongly influenced the friction performance. Moreover, Lin et al. (2015) adopted a microridge punch design that increased not only the friction between the sheet metal and the punch but also the pulling force of the microridges on the sheet metal, distributing the pulling force of the punch on the sheet metal to the microridges. This dispersed the pulling force at the nasal fillet of the punch on the sheet metal and substantially increased drawability.

Numerous researchers have studied the multistage deep drawing process. To confirm the reliability of the finite element analysis of the multistage rectangular cup drawing process, Ku et al. (2002) compared the results obtained from a simulation with the results of an experiment. In multistage elliptical cup deep drawing processes, Kim et al. (2001) used finite element inverse analysis to modify the original die design by enhancing the discrepancy in the thickness strain distribution for each intermediate die shape. With the aid of the finite element analysis, Kim et al. (2002) modified the rectangular cup with a large aspect ratio design to achieve smooth deformation and reduce the possibility of failure in the multistage drawing and ironing process. To lower the possibility of deformation failure, Kim and Hong (2007) designed and implemented a finite element simulation-based search algorithm to determine the optimal design variables of a multistage circular cup drawing process for a molybdenum sheet. To achieve a limiting drawing ratio of 9 for cylindrical brass cups, Faraji et al. (2010) used a limited number of annealing cycles during multistage deep drawing processes. Furthermore, Gau et al. (2013) conducted one deep drawing and two ironing stage experiments using 0.2 mm-thick 304 stainless steel sheets to investigate the effects of processing size. The experimental results showed that varying material annealing temperatures affected the forming heights.

The manufacture of cups with high aspect ratios involves numerous deep drawing stages, which results in high manufacturing costs. Therefore, the design goal of these processes is to reduce the number of stages. However, most published articles have focused on reducing the possibility of failure in the multistage deep drawing process.

This study applied the microridge punch in multistage deep drawing to reduce the number of processing stages. The original eight-stage deep forming process of a copper cylindrical cup with high aspect ratio was exemplified by introducing microridge punch designs to Stages 4 and 7 of the forming process, replacing the original punches and eliminating Stages 3 and 6. Subsequently, the feasibility of the new process was assessed using DEFORM 2D and confirmed through a physical experiment.

## Design and analysis of the cylindrical cup multistage forming process

**Fig. 1:**
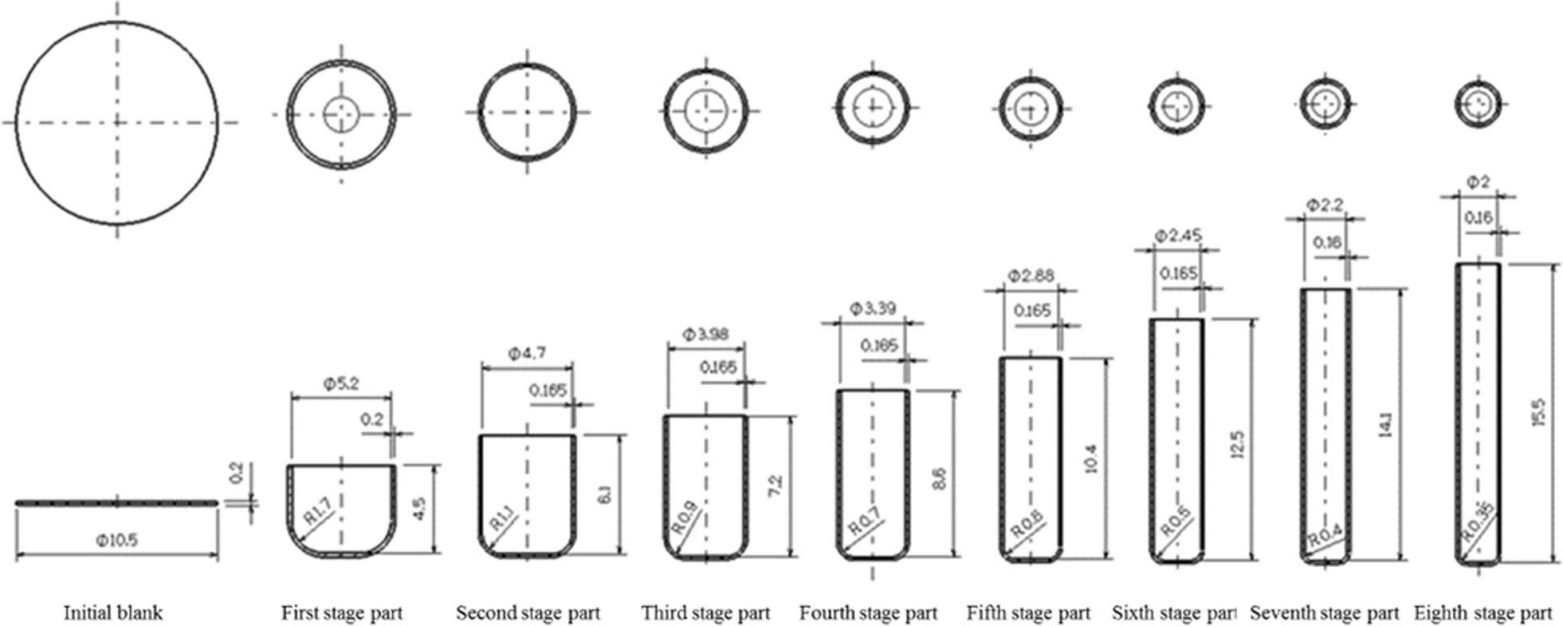
Schematic diagram of the original design parts and dimensions

The micro cylindrical cup multistage deep drawing process of 2.0 mm-diameter, 15 mm-deep, 0.16 mm-thick copper cups was investigated in this study. The relevant dimensions of the original component design are shown in Fig. 1.

### Original design

The geometric parameters of the multistage deep drawing dies comprised the punch diameter, punch nose radius, lower die shoulder radius, and die clearance. The main geometric parameters of the original designs used in each stage of the drawing process are shown in Table 1.

**Table 1:** Die design parameters and their values

	Original/modified design values				
	Punch diameter (mm)	Punch nose radius (mm)	Lower die shoulder radius (mm)	Clearance (mm)	Drawing ratio (%)
First stage	5.2/5.2	1.7/1.7	3.0/3.0	0.2/0.2	49.52/49.52
Second stage	4.7/4.7	1.1/1.1	3.0/3.0	0.165/0.165	90.38/90.38
Third stage	3.98/–	0.9/–	3.0/–	0.165/–	84.68/–
Fourth stage	3.39/3.39	0.7/0.7	3.0/3.0	0.165/0.165	85.18/72.13
Fifth stage	2.88/3.05	0.6/0.6	3.0/3.0	0.165/0.165	84.96/89.97
Sixth stage	2.45/–	0.5/–	3.0/–	0.165/–	85.07/–
Seventh	2.2/2.2	0.4/0.4	3.0/3.0	0.16/0.16	89.80/72.13
Eighth	2.0/2.0	0.35/0.35	3.0/3.0	0.16/0.16	90.91/90.91

**Fig. 2:**
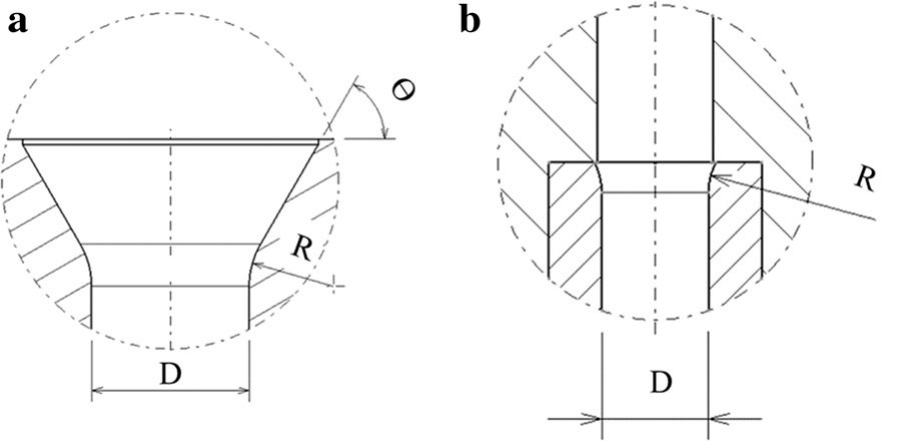
Schematic of lower die structure: **a** Stage 1, **b** other-stages

The lower die structure used in each stage of the forming process is shown in Fig. 2. To facilitate the sheet metal flowing into the lower die cavity and preventing wrinkle formation, the cavity of the die for Stage 1 was designed with intake openings, namely, a 60° intake angle (θ) and 3 mm intake corner radius (R). All processes in the subsequent stages adopted cylindrical guides in front of their cavities.

### Modified design

During the cylindrical cup drawing process, Stage 2 was completed, Stage 3 was eliminated, and Stage 4 (drawing) was executed.

**Fig. 3:**
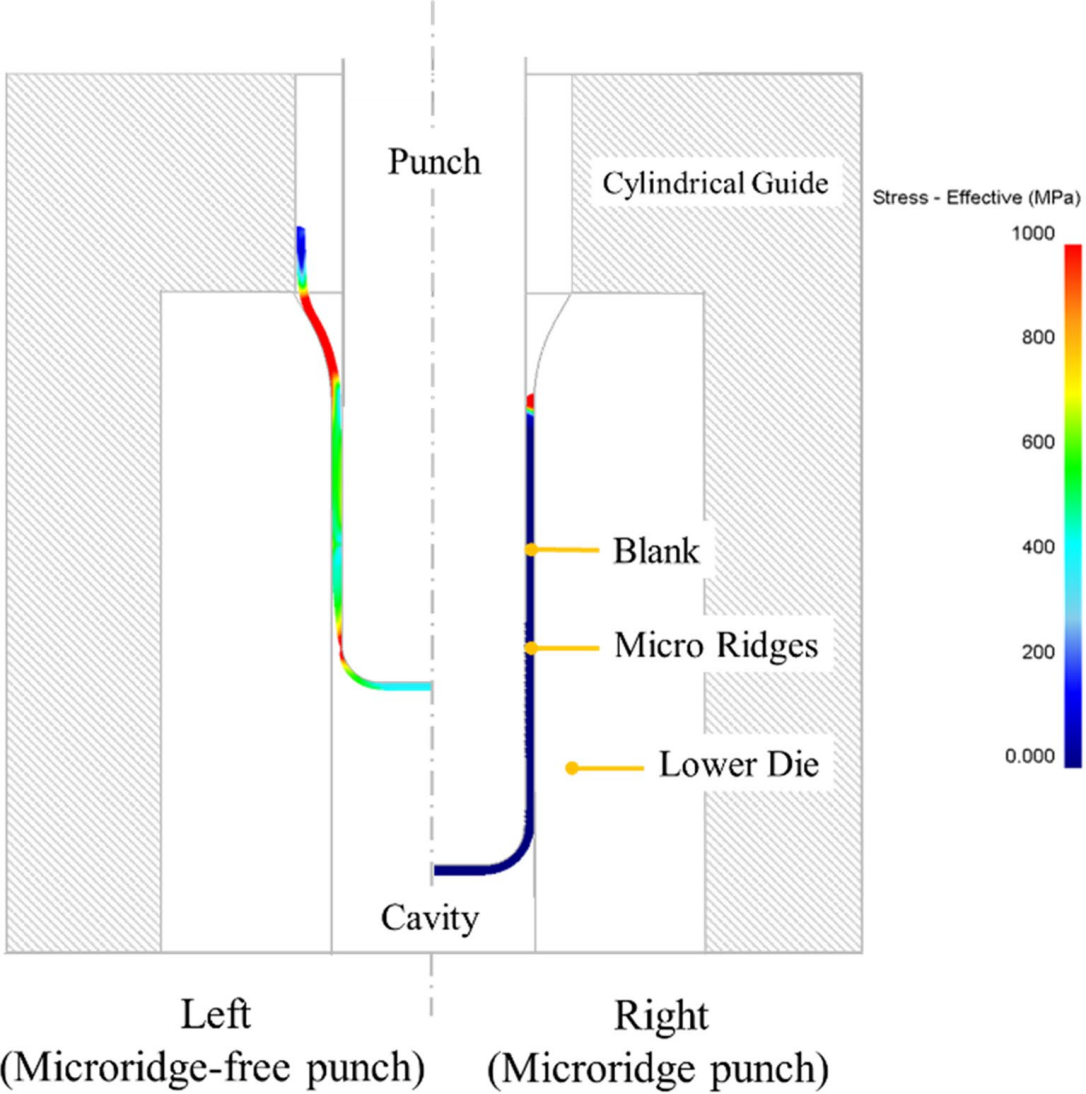
Deep drawing at Stage 4 of the modified design

At Stage 4, the process parts were positioned using the cylindrical guide, and the punch was moved downward at a uniform velocity to draw the blank. Figure 3 (left side) shows the result obtained when using a punch without microridges to deliver the drawing force from the nasal fillet to the blank. Accordingly, the highest stress of the blank occurred near the nasal fillet of the punch, and when this stress exceeded the tensile strength of the blank, the material cracked.

The right side of Fig. 3 shows several microridge rings on the sidewall surrounding the punch nose. These features increase the pulling force on the blank as well as the friction between the blank and punch by distributing the drawing force on the blank from the nasal fillet of the punch to the microridges. Consequently, the drawing force on the blank is no longer concentrated at the punch nose but is distributed through the punch nose and microridges. This slows sidewall thinning, preventing the occurrence of cracks.

The original design presents no cracks when Stages 3 and 4 are performed by using two microridge-free punches. However, in this example, Sage 3 is eliminated to follow the modified design. When Stage 4 is performed by using one microridge-free punch, the part will crack in this stage. In opposition, when Stage 4 is performed by using one microridge punch, the part presents no crack.

Lin et al. (2015) showed that a microridge punch increased deep drawing height by at least 100 % compared with a microridge-free punch in a single-stage deep-drawing process. This paper extends those results to explore the multistage deep drawing of two microridge punches. Specifically, a two-stage deep drawing using two microridge-free punches was replaced with a single-stage deep drawing using a microridge punch to reduce the number of deep drawing stages required.

The procedures involved in modifying the die design process are outlined in the following steps. First, CATIA was adopted to construct 3D models of the original eight-stage deep-drawing dies of copper cups according to the sizes of the dies at each deep-drawing stage. The 3D models were converted into axisymmetric 2D diagrams, and the diagrams were subsequently imported into DEFORM to analyze the formability of each stage of the original deep-drawing processes.

Next, the drawing stages that involved the use of a microridge punch were distributed evenly in the multistage forming processes. Moreover, because the microridge of the punch indents the inner wall of the cylindrical cup during deep drawing processes, the final drawing stage is designed using a microridge-free punch to decrease the indent in the final product. Hence, in the modified design, the microridge punch was designed in Stages 4 and 7 to replace the original punch, and Stages 3 and 6 were eliminated.

Finally, the design parameters (namely ridge height, ridge clearance, ridge nose radius, ridge shoulder radius, ridge-to-punch nose distance, and number of ridges) were specified for the microridge punch. An excessively high ridge height induces the ridges to be deeply pressed into the blank, whereas an extremely low ridge height causes the ridges to generate unsatisfactory drawing effects. Moreover, an extremely high ridge clearance results in the excessive distribution of the ridge drawing force to the blank. The minimum ridge clearance is limited to the thickness of the grinding blade. An extremely long ridge nose radius reduces the drawing force of the ridges; however, an excessively short ridge nose radius causes the ridge to be deeply pressed into the blank. In addition, an excessively long ridge shoulder radius induces the ridges to deliver limited drawing force. The minimum ridge shoulder radius is limited to the nasal fillet radius of the grinding blade. An extremely long ridge-to-punch nose distance results in increased thickness difference percentages, whereas an excessively short ridge-to-punch nose distance leads to changes in the appearance of components. In addition, an extremely high number of ridges increases the punch manufacturing costs, whereas an extremely low number of ridges reduces the drawing effect.

**Fig. 4:**
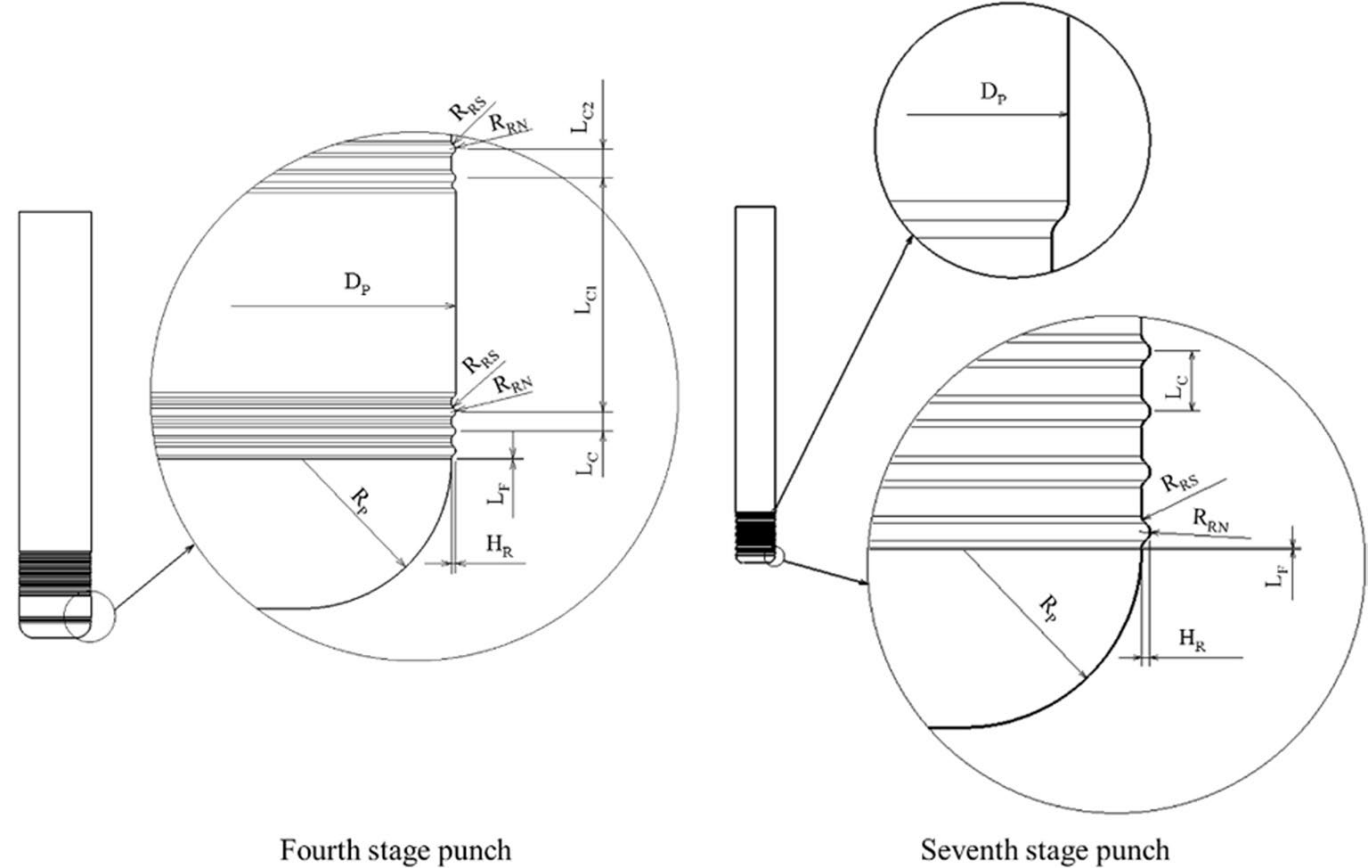
Schematic of the punch with microridges

The microridge punch design parameters are based on those used by Lin et al. (2015), although they were slightly adjusted, based on the comparison of DEFORM results with those of the original designs. The Stage 4 and 7 microridge punch designs are shown in Fig. 4, and the relevant design parameters are presented in Table 2. The modified die designs and their values are also shown in Table 1, indicating that the punch used in Stage 4 demonstrates two microridge segments. Because the results of the analysis revealed that the microridge in the middle did not contact the blank, the middle segment of the microridge was excluded from the design to reduce manufacturing costs.

**Table 2:** Dimensions of the punch with microridges

Dimension parameter	Fourth stage	Seventh stage
Punch diameter, D_P_ (mm)	3.39	2.2
Punch nose radius, R_P_ (mm)	0.7	0.4
Ridge height, H_R_ (mm)	0.019	0.019
Ridge clearance/ridge number		
L_C_ (mm)/Nc	0.09/3	0.135/18
L_C1_ (mm)/–	1.1/–	–
L_C2_ (mm)/Nc1	0.135/18	–
Ridge nose radius, R_RN_ (mm)	0.024	0.024
Ridge shoulder radius, R_RS_ (mm)	0.02	0.02
Ridge to punch nose distance, L_F_ (mm)	0.004	0.004

### Finite element analysis of copper cup drawing

This study investigated the copper cylindrical cup multistage deep drawing process by using a microridge punch to reduce the number of stages. During the deep drawing process, the microridges are pressed into the blanks to generate the drawing force. Therefore, this study adopted the commercial software DEFORM to analyze the multistage micro deep drawing process of copper cylindrical cups.

**Fig. 5:**
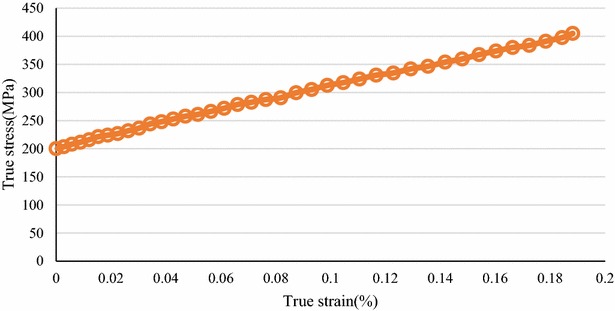
The true stress–strain curve of copper alloy C26800

The test material was 10.5 mm-diameter and 0.2 mm-thick rolled C26800 copper alloy sheet metal that was annealed for 1 h at 350 °C (composition shown in Table 3). In addition, the true stress–strain curve acquired from uniaxial tensile tests is shown in Fig. 5.

**Table 3:** Chemical composition of copper alloy C26800 (wt%)

Elements	Cu	Fe	Pb	Zn
C26800 (wt%)	64.0–68.5	<0.05	<0.09	Remainder

The FEM model mainly included the components of drawing punches with and without microridge features, a lower die, a blank holder, and a blank. The die components and products exhibited axial symmetry; therefore, 2D axisymmetric elements were adopted in the simulations. The geometric parameters of the multistage deep drawing dies comprised the punch diameter, punch nose radius, lower die shoulder radius, and die clearance. The relevant parameters and values are shown in Table 1. In addition, the modified microridge punch dimensions for Stages 4 and 7 are shown in Fig. 4 and Table 2.

**Fig. 6:**
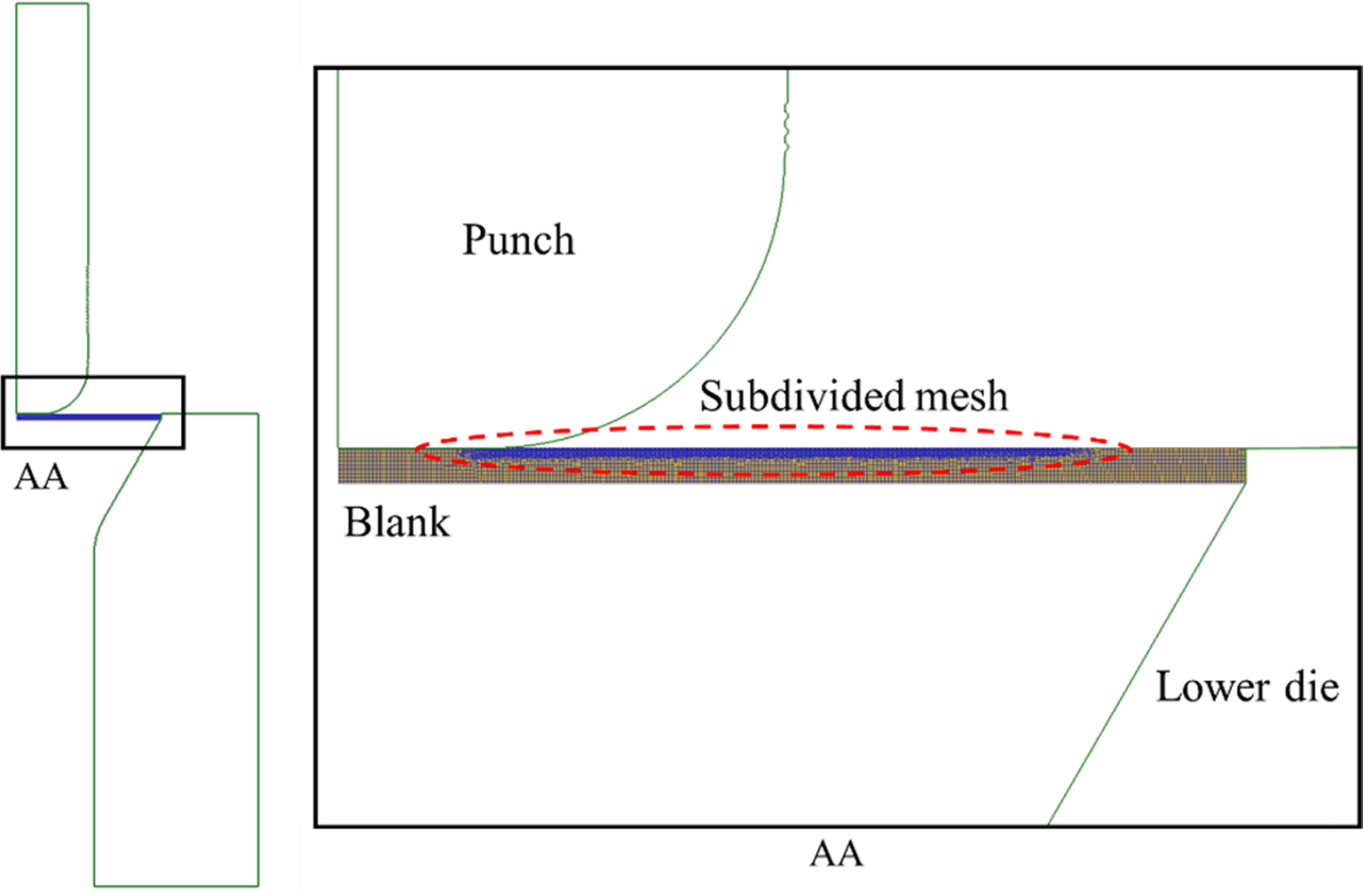
Simulation model of the micro deep drawing process

In this simulation, the die (comprising the blank-holder, lower-die, and punch) was assumed to be a rigid body in which the geometric dimensions were unaffected by external force and temperature variations. The blank was assumed to be isotropic and formed from a rigid-plastic material. When meshing the 2D sectional surface, five layers of surface mesh on the top surface of the blank were refined up to 0.004 mm × 0.004 mm to demonstrate the microridge features accurately, as shown in Fig. 6. The coarse mesh in the unrefined areas had a mesh size of 0.02 mm × 0.02 mm. The Coulomb model was employed to simulate the die-blank and punch-blank friction conditions, using constant coefficients of 0.1 and 0.3, respectively. In the simulated motion, the punch completed the forming process while traveling downward at a uniform velocity of 10 mm/min.

## Multistage micro deep drawing experiment

### Experimental equipment

**Fig. 7:**
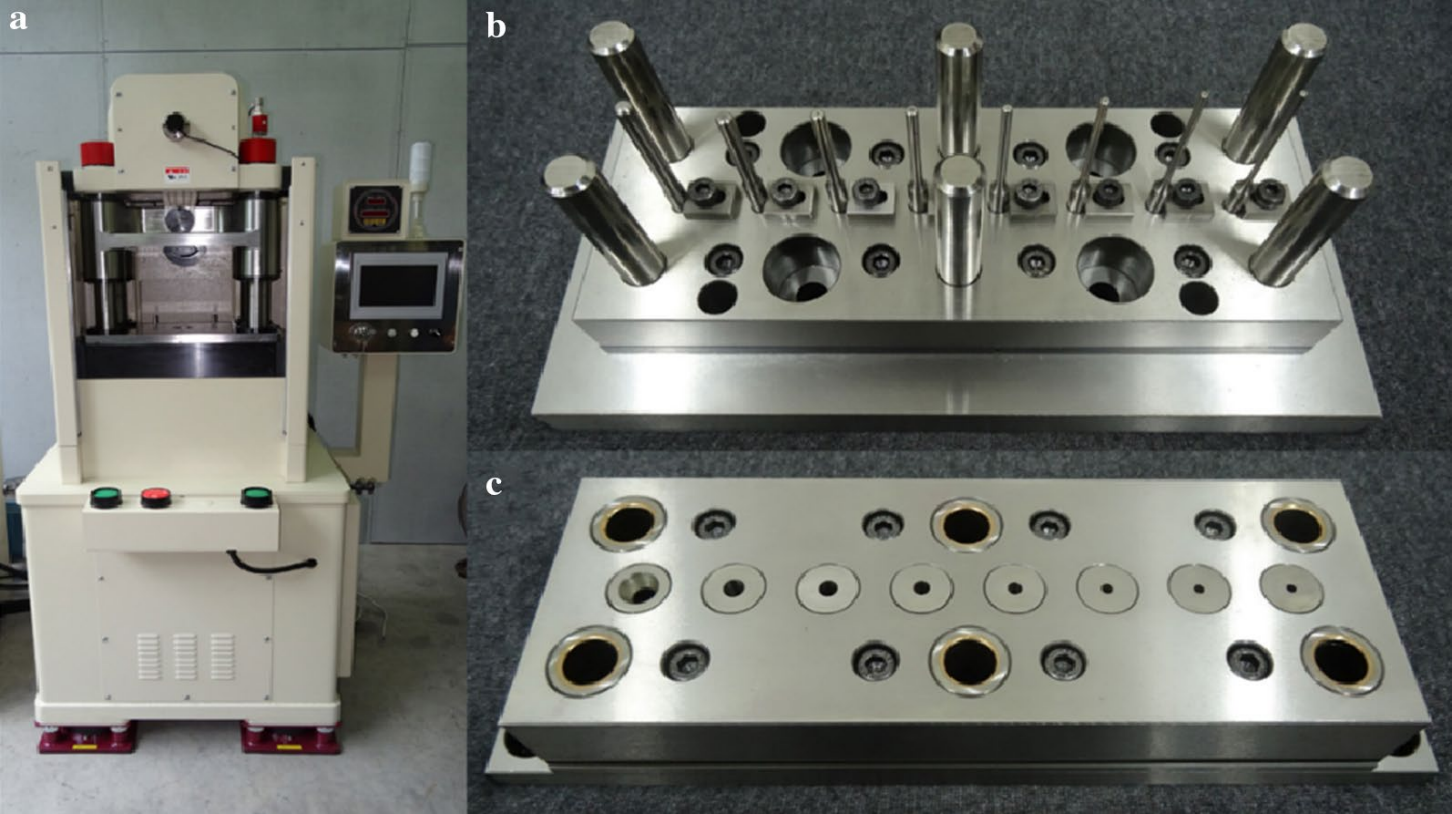
Experimental apparatus: **a** 3-tons precision press machine, **b** upper experimental die set, **c** lower experimental die set

**Fig. 8:**
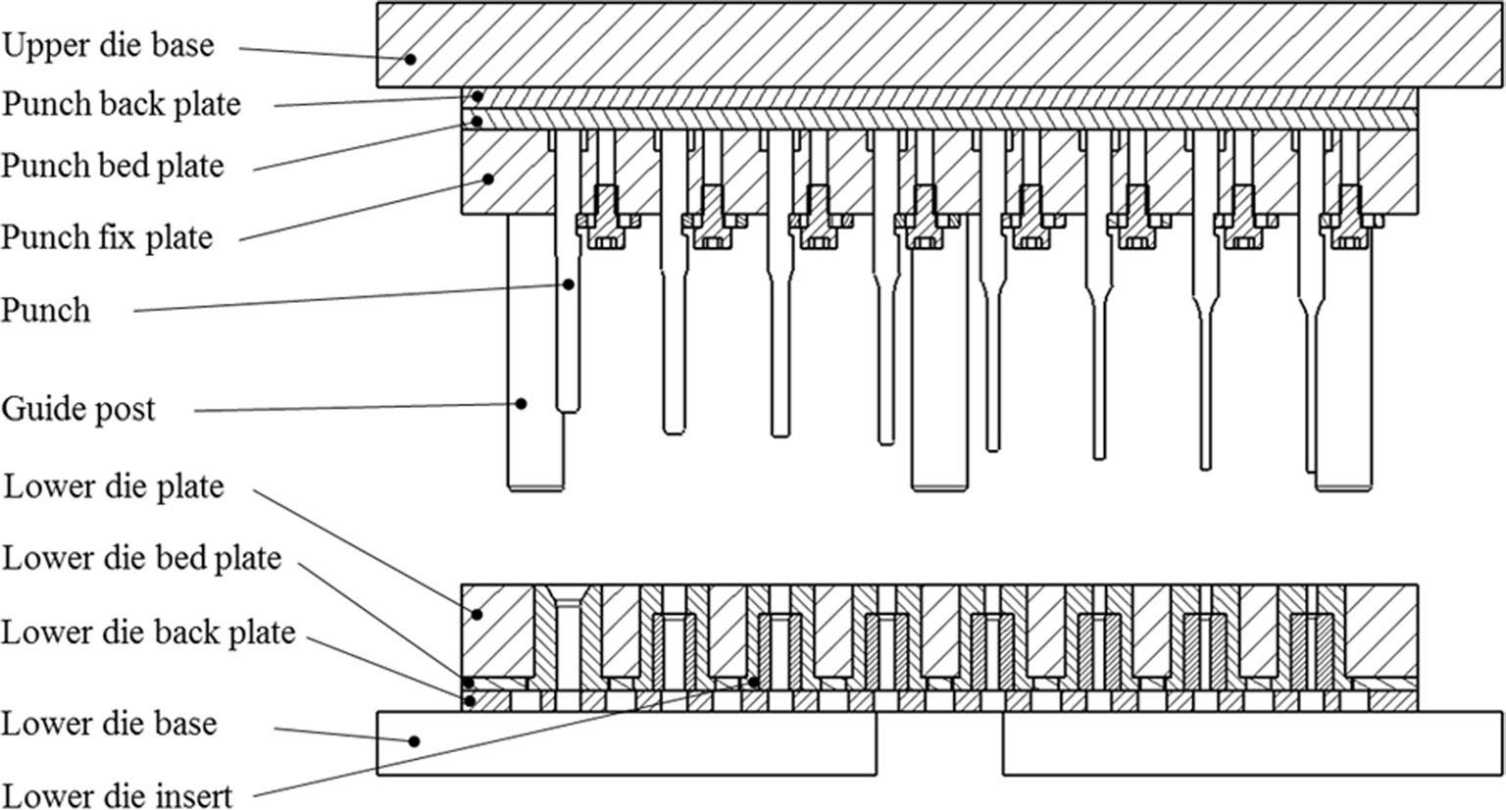
Multi-stage deep drawing die

The multistage micro deep drawing experiment used experimental dies and a 3-ton precision press machine, which are shown in Fig. 7. The experimental dies for the copper cylindrical cups are shown in Fig. 8. These were designed according to the original eight-stage micro-forming process.

### Experimental procedure

A section of 0.2 mm-thick rolled C26800 copper alloy sheet metal was used as the test material. Cylindrical blanking dies were employed to cut 10.5 mm-diameter cylindrical blanks from the test material. To obtain quality blanks and prevent deckle edges, the clearance between the lower die and cutting punch was 1.5 % of the material’s thickness.

To verify if the microridge punch could reduce the number of forming stages, this experiment first adopted the eight-stage process, using microridge-free drawing punches. Next, a six-stage process was adopted by introducing a microridge punch to Stages 4 and 7 and eliminating Stages 3 and 6.

The multistage micro deep forming test was conducted at room temperature through the following steps: (a) The cylindrical blanks were wiped clean. (b) The blanks were positioned on the V-shaped conical openings. (c) The deep forming process was conducted using punches with and without microridge designs at a forming velocity of 10 mm/min. (d) The semifinished blank products were transferred to the next lower die cavity and positioned on the cylindrical guide. (e) Steps (c) and (d) were repeated until product completion. To reduce friction coefficients, relevant components in each experimental forming stage were adequately lubricated. Additionally, all experiments were repeated at least twice to ensure reproducibility.

## Results and discussion

### Deformed shapes

**Fig. 9:**
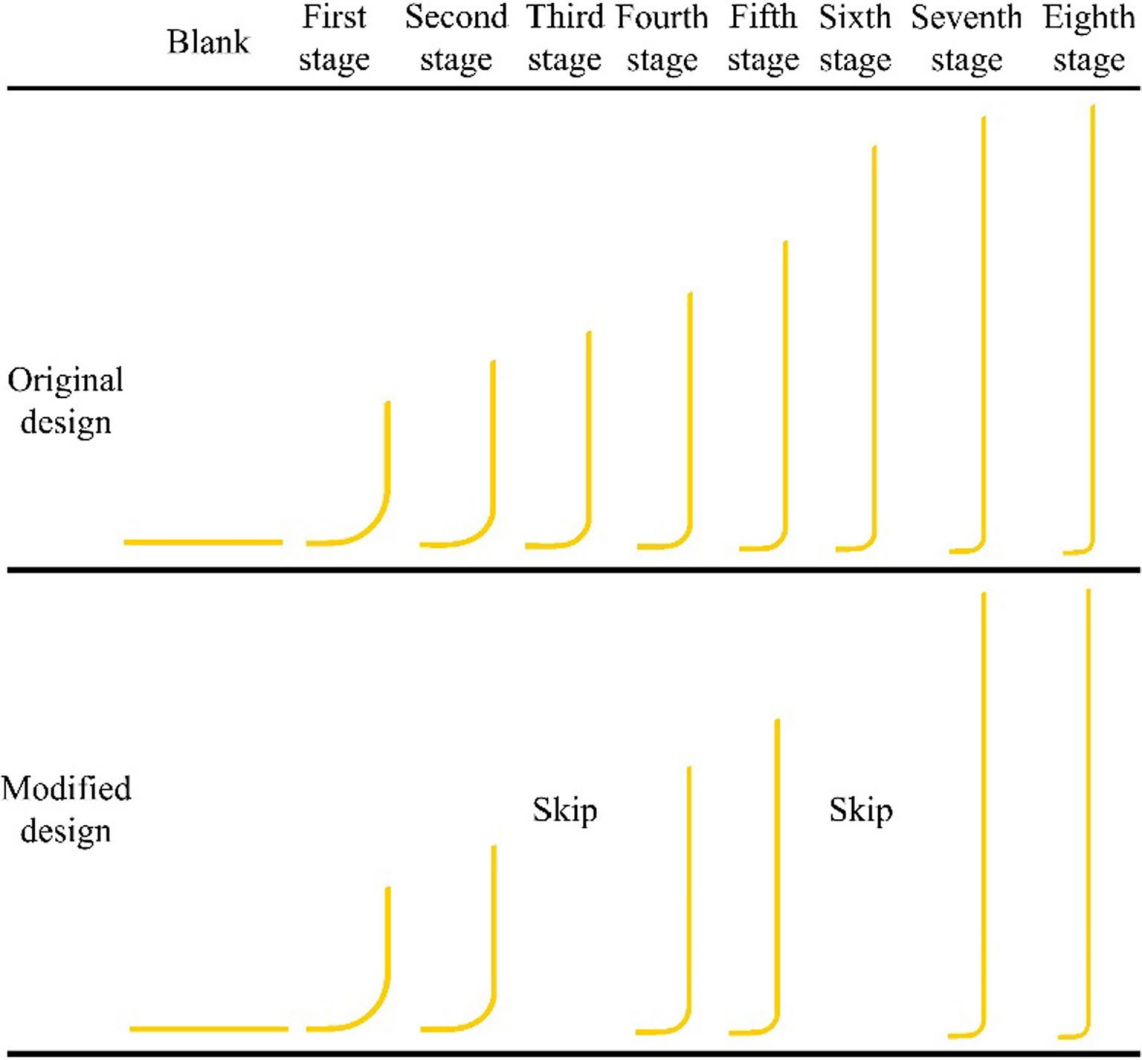
Simulation results of the copper cup

The simulation results of the original and modified copper cup deep drawing designs are shown in Fig. 9. During deep drawing, the thinning of the components of each stage varies, and drawing-induced cracks can occur in the thinnest parts of the components. Accordingly, the thickness difference percentage can be defined as (blank thickness − minimum thickness of drawn part)/(blank thickness) × 100 %, and is used as the criterion for determining the occurrence of cracks on components during drawing. The thickness difference percentages for each stage are presented in Table 4, and the corresponding thickness difference percentages of the original and modified designs were consistent and remained at approximately 30 %.

**Table 4:** Simulation results of the copper cup

Stage	Original/Modified design		
	Forming height (mm)	Thickness difference percentage (%)	Maximum deformed load (N)
First stage	4.5/4.5	10.85/10.85	2816/2816
Second stage	5.9/5.9	13.73/13.73	3147/3147
Third stage	7.1/–	16.58/–	1103/–
Fourth stage	8.5/8.6	20.07/19.68	1495/2326
Fifth stage	10.2/9.8	23.08/23.55	1646/1266
Sixth stage	13.4/–	25.71/–	2414/–
Seventh stage	14.4/14.2	28.35/25.50	918/2435
Eighth stage	15.7/15.5	29.25/30.53	657/730

**Fig. 10:**
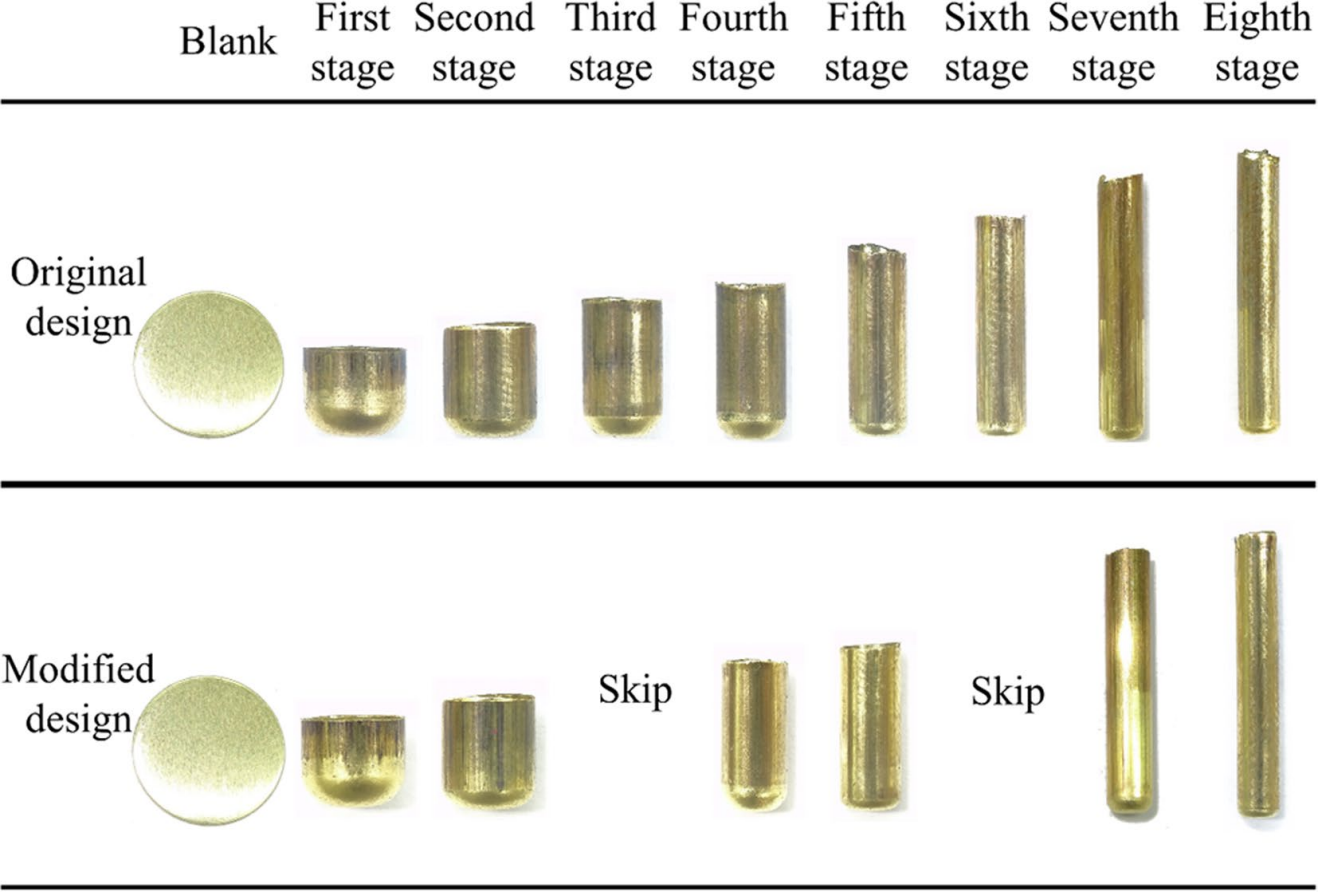
Experimental results of the copper cup

Figure 10 shows the results of the original and modified copper cup multistage drawing experiments. Slight earing was observed in the finished products from each stage. These features were caused by the die process, assembly tolerance, and experimental blank positioning errors. The experimental products of the original and modified processes exhibited identical shapes and heights through the corresponding forming stages. Additionally, no surface flaws were generated at Stages 4 and 7 of the modified microridge forming process.

### Punch force

**Fig. 11:**
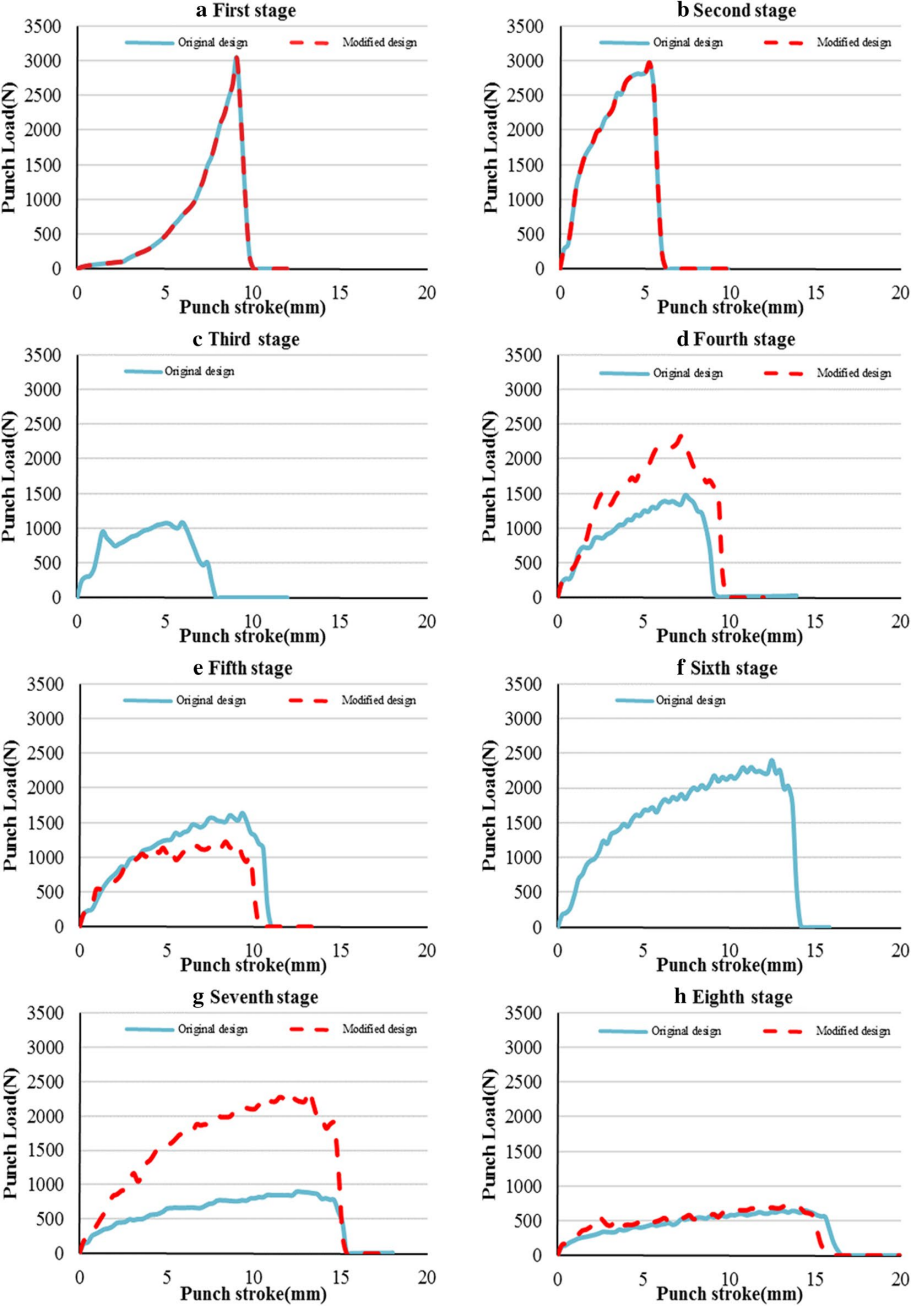
The simulated forming loads of the drawn copper cup. **a** First stage original/modified design. **b** Second stage original/modified design. **c** Third stage original design. **d** Fourth stage original/modified design. **e** Fifth stage original/modified design. **f** Sixth stage original design. **g** Seventh stage original/modified design. **h** Eighth stage original/modified design

 Figure 11 shows the numerical prediction of the punch force evolution with its displacement, for each specific stage, of the original and modified copper cup designs. Because the die structure and size of Stages 1 and 2 in the modified copper cup design are based on those of the original design, the punch load curves for both designs are identical. Moreover, the rising rate of the punch load of Stage 1 increased with the punch stroke, whereas those of the other stages decreased, because the lower die structure of Stage 1 differed from those of the subsequent stages (Fig. 2).

### Thickness of the deformed parts

Because product thickness was low, a wire electric discharge machine was used for sectional processing in each product-forming stage to prevent deformation. The wire electric discharge machine hardened the product surface, affecting measurement quality. Therefore, the hardened surface layer was removed using sandpaper before obtaining relevant measurements. To ensure measurement precision, a confocal microscope was used to measure the cross-sectional thickness of the products for each forming stage.

**Fig. 12:**
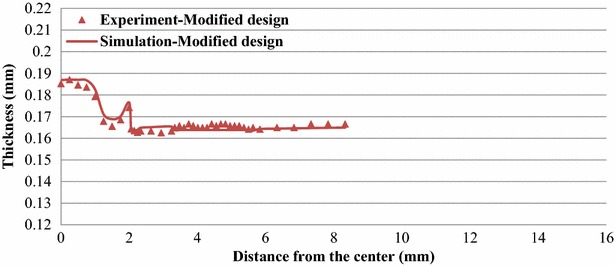
Thickness distribution at Stage 4 of the modified design

**Fig. 13:**
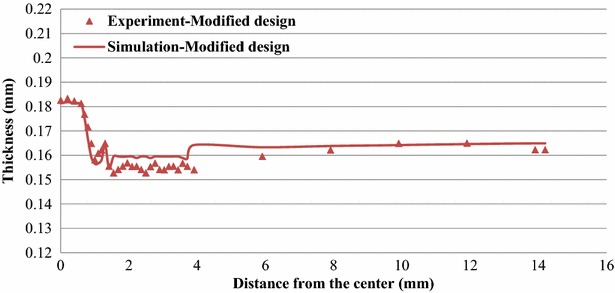
Thickness distribution at Stage 7 of the modified design

**Fig. 14:**
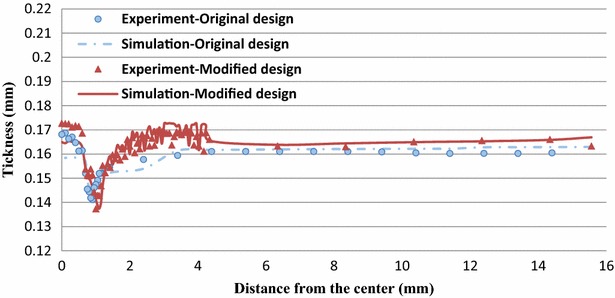
Thickness distribution at Stage 8 of the original and modified design

Figures 12 and 13 display the thickness distributions of the simulated and experimental forming products for Stages 4 and 7 of the modified designs. Figure 14 shows the simulated and experimental results for the thickness distributions at the Stage 8 of the original and modified designs. The simulated thickness distributions were consistent with the experimental results. Furthermore, the experimental product exhibited severe thinning near the punch nose radius and dents caused by the first ring of the microridges. These features were also identified in the simulation results.

**Fig. 15:**
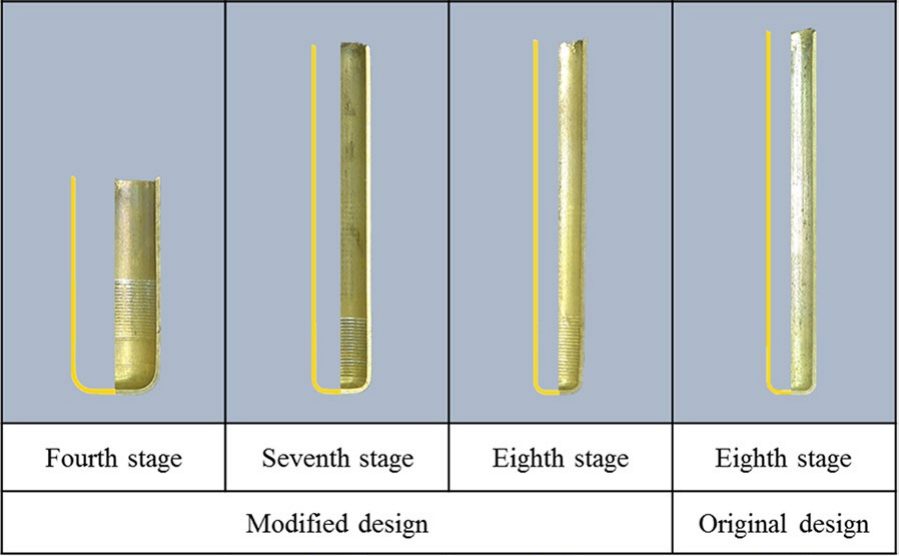
Comparison of cross-sections of the simulated and experimental results

Figure 15 shows the simulated and experimental cross-sectional shapes from Stages 4 and 7 of the modified design, and Stage 8 of the original and modified designs. The simulated results in the cross-sectional deformation were identical to the experimental results. At Stage 5, 7, and 8 of the modified design, dents were observed at the contact ring between the blank and the microridge on the punch. However, no flaws were discovered on the inside surface at Stage 8 of the original design.

## Conclusions

Microridge punches were designed to decrease the number of stages in the multistage deep drawing process of the high aspect ratio cylindrical cups with the aid of the finite element method. Explicit rigid-plastic finite element analysis with 2D axisymmetric solid elements was performed to calculate the thickness difference percentage in the initial design during the deep drawing process. Guideline of the process design modification was proposed to reduce the number of deep drawing stages at similar thickness difference percentages. The modified design proposed that a two-stage deep drawing using two microridge-free punches was replaced with a single-stage deep drawing using a microridge punch. The deep-drawing process used microridge punches evenly distributed throughout the drawing stages to decrease the indent in the final product. The design parameters of the microridge punches were set based on the comparison of the simulated results with those of the original designs. The modified design was examined by the simulated and experimental results.

A micro multistage deep drawing experiment was conducted in this study to deform 0.2 mm-thick and 10.5 mm-radius C26800 copper alloy sheet metal plates into 2.0 mm-diameter, 15 mm-deep, and 0.16 mm-thick copper cups. Experiments were conducted to compare the forming processes by using a punch nose with and without surface microridge designs. The simulated and experimental results are summarized as follows:

The traditional punch requires eight stages of deep drawing. The modified process involves replacing the microridge-free punches in the original Stages 3–4 and 6–7 by introducing microridge designs in Stages 4 and 7. Specifically, a single processing stage using microridge punch designs replaces two processing stages without using microridge punch designs. Consequently, the microridge designs shorten the eight-stage deep drawing process to a six-stage process.

The experimental testing was conducted according to the simulation results. The comparison showed the thickness distributions demonstrated consistency. Therefore, the commercial forming analysis software accurately simulated the multistage micro deep forming process of the microridge punch designs.

According to the simulated and experimental results, the Stage 8 cups with original and modified designs yielded similar final thickness distributions with maximum thickness difference percentages of approximately 30 %.
